# Magnetic Resonance Imaging in the Management of Women with Low-Risk Early-Stage Cervical Cancer: A Narrative Review

**DOI:** 10.3390/diagnostics15080985

**Published:** 2025-04-12

**Authors:** Sara Boemi, Giada Guagliardo, Sara Pasi, Martina Somma, Alessia Pagana, Maria Teresa Bruno

**Affiliations:** 1 Department of Radiology, Fondazione IRCCS Cà Granda, University of Milan, 20122 Milano, Italy; 2 Department of Experimental and Clinical Medicine, University of Catanzaro, 88100 Catanzaro, Italy; 3 Department of General Surgery and Medical-Surgical Specialties, Gynecological Clinic, University of Catania, 95123 Catania, Italymt.bruno@unict.it (M.T.B.)

**Keywords:** magnetic resonance imaging, early cervical cancer, parametrial resection, sentinel lymph node dissection, ultrastaging, radical hysterectomy

## Abstract

Radical hysterectomy continues to be the standard treatment for early-stage cervical cancer. Accurate staging plays an important role in the management of cervical cancer, and preoperative imaging is indispensable to ensure appropriate stage assignment and to identify the surgical patient. Many studies have highlighted the need to consider the low-risk patient in a markedly different way from the intermediate- or high-risk patient. The aim of this study is to highlight the most urgent divergences to be addressed in low-risk early cervical cancer patients, such as reducing the radicality of the surgery, avoiding parametriectomy with tumors smaller than 2 cm, replacing bilateral lymphadenectomy with sentinel lymph node dissection, persistence of MIS instead of laparotomy, and preserving fertility. All this evidence is the result of the progress that has been made in recent decades in the early diagnosis, staging, and treatment of cervical cancer, made possible not only by the ever-increasing experience of gynecological oncologists but above all by the increasingly sophisticated imaging techniques that support the work of the clinician.

## 1. Introduction

Cervical cancer is the first neoplasia for which an infectious cause has been recognized, with persistent HPV (Human Papillomavirus) infection having been recognized as an independent cause of its onset [1]. HPV infection, transmitted sexually, is largely preventable. Screening programs for the population at risk and primary prevention with the HPV vaccine have contributed over the years to reducing its incidence [2,3,4,5]. Despite these preventive measures, approximately 604,000 new cases and 342,000 deaths were reported in 2020, making cervical cancer the fourth most common cancer among women worldwide [6].

The World Health Organization (WHO) has developed a global HPV eradication program, establishing an incidence limit of 4 cases per 100,000 women as a criteria +for pragmatic HPV elimination [7]. The WHO’s “90-70-90” target aims to ensure that 90% of girls are fully vaccinated against HPV by the age of 15, that 70% of women are screened with a high-throughput test by the age of 35 and again by the age of 45, and that 90% of women with precancerous lesions and invasive cervical cancer receive appropriate treatment. This target should be achieved by 2030 for countries to be on the path to eliminate cervical cancer. In the last few years, there has been an increase in the incidence of preneoplastic lesions, such as high-grade squamous intraepithelial lesions (HSILs) and adenocarcinoma in situ (AIS), and in the diagnosis of early-stage cervical carcinoma thanks to the widespread availability and diffusion in Western countries of early diagnosis, staging, and treatment techniques, which have led to an increase in survival and quality of life [8,9,10,11,12,13,14].

Macroscopically, cervical carcinoma may present an exophytic form, typical in young women, or an infiltrating or endocervical form with a barrel shape determined by the introflexion of the squamocolumnar junction (SCJ) into the cervix of postmenopausal women. Cervical cancer is a malignant neoplasm that grows at a relatively slow rate. However, it demonstrates early lateral extension along the parametrium and uterosacral ligaments. As such, the tumor tends to involve the mucosa of the lateral vaginal fornixes and may spread to the remaining part of the vagina. The rectum and bladder may also be involved, the latter more easily, because the anterior fornix is narrower and the vesicocervical septum is thinner than the vaginorectal septum. Rectal infiltration can be appreciated with combined rectal and vaginal exploration, presenting as an irregular thickening of the rectovaginal space and with a fixity of the rectal mucosa. The tumor spreads via the lymphatic system through the parametrium where the lymphatic vessels run. The tumor then reaches the parametrial, pelvic, para-aortic and para-caval lymph nodes. The pelvic lymph node stations that are usually the first to be involved are, by frequency, the external iliac, the obturator, the hypogastric (or internal iliac), and the common iliac. Bloodstream spread most frequently involves the lung, liver, intestine, and bone. The survival of cervical cancer patients is strongly associated with the extent of disease, both local and distant. Previous studies have shown that tumors larger than 2 cm are more frequently associated with parametrial involvement and lymph node metastasis [14,15]. Therefore, variables, such as tumor size, stromal invasion, and LVSI, are considered prognostic risk factors [16]. Precise tumor staging at diagnosis is fundamental for adequate treatment planning. Tumors that are under-staged at surgery and, therefore, require adjuvant chemoradiation have been shown to be associated with increased morbidity. Therefore, careful selection of surgical candidates is essential [16].

One of the most widely used classification systems for cervical cancer is the International Federation of Gynecology and Obstetrics (FIGO); until 2018, it was exclusively clinical, and lymph node status was not studied, despite its important impact on prognosis and management [17,18]. Although not implemented in the 2009 FIGO guidelines, nuclear magnetic resonance imaging (NMR), for the high-resolution study of soft tissues, has been used in the study of cervical carcinoma since the 2000s and has been shown to be very accurate in determining tumor size, parametrial invasion, and lymph node metastases. In a recent review, the overall accuracy of MRI in the evaluation of lymph node metastases was reported to be adequate (73–90%) with a high specificity (75–91%) and NPV (71–96%), but poor sensitivity (52–75%) and PPV (52–75%). Other studies reported the overall accuracy values of MRI for assessing parametrial, vaginal, and lymph node status, which were 65.8%, 79.4%, and 79.4%, respectively [19,20].

These peculiarities of MRI and imaging in general have led to the update of the FIGO 2018 classification by adding stages that emphasize the primary tumor size and the status of the lymph nodes: this has made the FIGO 2018 staging more clinically relevant [4,21,22]. Furthermore, the introduction of the study of the lymph node status has made the FIGO 2018 classification comparable with the TNM classification of the American Joint Committee on Cancer (9 AJCC TNM) version 2021 [23]. Preoperative MRI is essential for an accurate assessment of tumor size [24]. The new FIGO 2018 guidelines separate the T1B1, T1B2, and T1B3 stages based on the tumor size [16]. Tumors smaller than 2 cm are classified as IB1 and can be treated surgically by simple or radical hysterectomy; tumors between 2 cm and 4 cm are classified as IB2 and are treated depending on the degree of stromal invasion with radical hysterectomy or chemoradiotherapy; patients with tumors larger than 4 cm are classified as IB3 and are treated primarily with chemoradiotherapy.

In the new FIGO classification, a new FIGO stage for pelvic (C1) and para-aortic (C2) lymph nodes was introduced, namely stage IIIC. All patients with positive lymph nodes, regardless of the diameter of the primary tumor, belong to this new stage. Micrometastases are included in stage IIIC. From this perspective, it is very important to have a preoperative assessment of lymph node status. In early-stage disease with suspicious lymph nodes on MRI, PET is recommended to assess the lymph node status; intraoperative evaluation of sentinel lymph nodes on both sides of the pelvis and/or any suspicious lymph nodes is mandatory. A radical surgical approach can only be performed if the nodes are negative. The rest of the staging relies on the size of the primary tumor.

The use of MRI increases diagnostic accuracy and allows recording the following characteristics of the tumor: size (all three dimensions), location (endo/ectocervix), distance from the internal os (mm), depth of infiltration of the cervical stroma (superficial: <5 mm, deep: more than two-thirds of the total thickness of the cervical stroma, full-thickness stromal invasion: involvement of the serosa), cranial extension of the tumor to the uterine body, infiltration of the vagina and possible extension to its lower third, parametrial infiltration, extension of the tumor to the pelvic wall, infiltration of the rectovaginal septum, infiltration of the vesicouterine septum, infiltration of the rectal mucosa, hydroureteronephrosis, lymph node metastases, and distant metastases. The resulting instrumental staging allows for accurate treatment planning that can range from immediate surgical treatment with the possibility of a fertility-sparing approach (in the presence of a tumor < 2 cm, distance from the OUI > 1 cm, and length of the cervix > 2.5 cm), to neoadjuvant therapy with chemo- or chemoradiotherapy up to exclusive radiotherapy treatment. But, above all, it allows preoperative differentiation between early cancer and locally advanced disease, necessary to avoid the combination of radical surgery and chemoradiotherapy, associated with serious comorbidities (lymphedema, lymphocystis, gastrointestinal morbidities, and genitourinary complications).

Early-stage cervical cancer refers to cancer confined to the cervix, with a diameter of less than 2 cm, no parametrial invasion, and negative lymph nodes, typically treated with primary surgery. Locally advanced cervical cancer (LACC) is characterized by invasive carcinoma greater than 4 cm in greatest dimension and/or tumor extension beyond the uterus (stages IB3–IVA). LACC is treated with concurrent chemoradiation (RCT), typically involving cisplatin-based chemotherapy and external beam radiotherapy (EBRT), followed by intracavitary brachytherapy (ICBT).

The aim of this review is to explore the role of MRI in diagnosing and determining the treatment stratification for patients with early cervical cancer.

## 2. Materials and Methods

To identify relevant clinical trials, a comprehensive literature search was conducted by several authors in different databases. We searched for relevant articles in the three data-bases PubMed/MEDLINE, Scopus, and Web of Science. All articles that meet the following key criteria were selected: “cervical cancer”, “cervical cancer staging”, “sentinel lymph node”, “magnetic resonance imaging”, “fertility sparing”, “positron emission tomography (PET-CT)”, “parametriectomy”, “MIS”, and “minimally invasive surgery”.

The selection of articles was subject to a consistent review and assessment in order to identify studies that were potentially relevant to the objectives of this review.

The main inclusion criteria were as follows: (1) articles in English, (2) original studies investigating the role of radiological imaging in CC, (3) studies examining the role of PET-CT and PET-MRI, (4) studies enrolling patients with low-risk early cervical cancer, (5) studies reporting survival data (overall, recurrence rate, disease-free survival), (6) studies comparing oncological outcomes between SH and RH in patients with early cervical cancer, and (7) studies including women undergoing fertility-sparing treatments.

This review excluded editorials, case reports, and letters. Studies that met the inclusion criteria were further analyzed, and relevant data were extracted and evaluated for each article. Any discordances between investigators were resolved through a consensus approach.

## 3. Results

The search generated 130 relevant articles. It was restricted to articles published between 1998 and 2023. In the beginning, a total of 111 articles were identified as potentially relevant for the review. Ultimately, 72 articles were included, which met the inclusion criteria [8,10,11,12,14,15,16,17,18,19,20,21,22,23,24,25,26,27,28,29,30,31,32,33,34,35,36,37,38,39,40,41,42,43,44,45,46,47,48,49,50,51,52,53,54,55,56,57,58,59,60,61,62,63,64,65,66,67,68,69,70,71,72,73,74,75,76,77,78].

## 4. Magnetic Resonance Imaging and Treatment of Early Cervical Cancer

### 4.1. Magnetic Resonance Imaging: Protocol

MRI is the most suitable technic for the evaluation of cervical cancer due to its ability to ensure high-contrast resolution for the study of soft tissue, significantly superior to CT [25]; MRI is an essential method to ensure correct diagnosis, staging, and prognosis. It is used not only in staging but also in the evaluation of treatment response as well as in the monitoring of radiotherapy and during post-treatment follow-up.

MRI scans should be conducted on a machine with 1.5 T or 3 T field strength, with the latter clearly being superior due to its much better signal/noise ratio, using variable phase body coils.

To reduce bowel evacuation artifacts, an antiperistaltic, such as butylscopalamine bromide (Buscopan), can be administered intramuscularly shortly before the examination (considering contraindications, such as glaucoma); the bladder must be semi-full to ensure correct positioning of the uterus and, if an advanced stage of the disease with infiltration of the vaginal and/or rectal walls is suspected, intracavitary administration of sterile aqueous gel is performed to facilitate the distinction of the cavities and to effectively study the infiltration of the wall. Magnetic resonance imaging must be executed before biopsy or at least 10 days after it to prevent false positive results caused by local inflammation [26].

### 4.2. T2-Weighted Images (T2WI)

**Figure 1:**
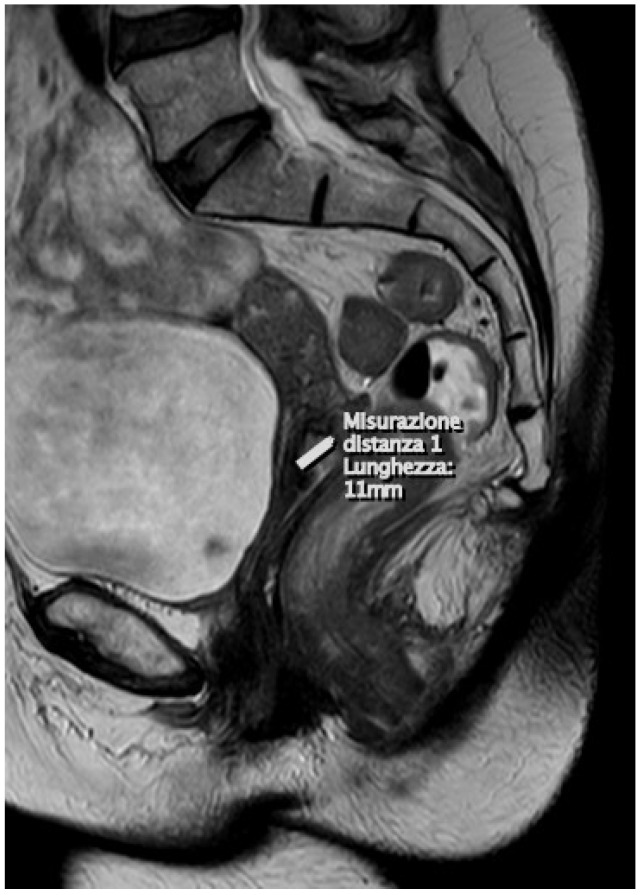
At the uterine cervix, the presence of a solid infiltrating neoplastic lesion of the dimensions of (DAP × DT × DL) 11 × 21 × 16 mm, respectively, is confirmed, which invades the stroma (>3 mm) from the mucosa of the middle portion of the endocervical canal. The aforementioned formation appears hyperintense in T2.

The standard MRI protocol for cervical cancer evaluation includes an initial sagittal T2WI-weighted sequence with a large field of view (FOV). This is used to create another image with a small FOV at a high resolution and perpendicular to the long axis of the cervix in order to improve the assessment of the local extension of the tumor and the more or less present invasion of the parametrium; therefore, T2-weighted sequences are acquired and oriented in the three planes (para-axial, para-coronal, and para-sagittal). These are crucial sequences to assess the accurate size of the tumor and the potential infiltration of nearby organ walls. The oblique coronal plane effectively evaluates the invasion of the parametrium [27] (Figure 1).

### 4.3. Diffusion-Weighted Images (DWI)

DWI is another functional magnetic resonance imaging sequence that is increasingly being used in the study of cervical cancer [28]. DWI provides information on the microstructure of the tumor, which appears hypercellular, so there is less movement of water within the tumor tissue [8]. It allows us to distinguish between water molecules with free diffusion (as in cerebrospinal fluid) and those with restricted diffusion (by cell membranes, fibers, macromolecules, etc.). Since the tumor tissue is hypercellular, the diffusion of water is reduced, which makes this technique particularly interesting in the study of tumors. A quantitative assessment of the diffusion capabilities of water molecules in the analyzed tissue is obtained using the apparent diffusion coefficient (ADC), expressed in square millimeters per second. This value reflects the exponential decrease in tissue signal intensity with increasing diffusion weighting (b values) [10,29].

Cervical cancer tissue typically exhibits restricted diffusion, showing high signal intensity for the primary tumor and metastatic lymph nodes on high b-value DWI, along with corresponding low signal intensity on the ADC map. In practice, DWI highlights malignant lesions with high signal intensity but can obscure normal anatomical structures and the precise location of the tumor. Combining conventional anatomical images, such as T2WI, with DWI through fusion software enhances the recognition and detection of carcinoma. DWI-weighted sequences and corresponding ADC maps are always acquired based on the axial and sagittal planes of the corresponding T2WI-weighted “anatomical maps”. The DWI technique has proven to be highly sensitive in detecting parametrial invasion (81%) and lymph node metastasis (86%) [30,31]. Additionally, incorporating DWI has been shown to increase reader confidence and improve tumor delineation, particularly among less experienced radiologists. Furthermore, the maximum tumor dimensions measured from DWI are virtually identical to those derived from conventional imaging series [32].

The most recent ESUR guidelines emphasize that T2WI and DWI sequences, ideally combined in the acquisition plane, are crucial for initial staging [33].

DWI facilitates lymph node detection, but several studies have highlighted how DWI-weighted sequences can be misleading since both pathological and physiological lymph nodes present a restriction of the diffusion signal, hence the importance of supporting the DWI analysis with strictly anatomical sequences obtained by T1WI and T2WI weighting; a very important criterion in this case will be the evaluation of the short axis dimensions of the lymph node, taking into account the normality of the 15 mm short axis of the inguinal lymph nodes and the pathology of the 8 mm short axis in case of evaluation of the obturators [34]. For the evaluation of lymph nodes, PET is now considered the best option due to its higher sensitivity and specificity (73% and 98%, respectively) when compared to MRI (56% and 93%, respectively) and CT (58% and 92%, respectively) [35].

MRI is now the method that allows researchers to define with greater accuracy the size of the tumor, its location (exophytic or endocervical), and its parametrial infiltration (Table 1).

### 4.4. Surgical Therapy

Currently, radical hysterectomy (RH) and chemoradiotherapy (CRT) are the most frequent treatment modalities for patients with early-stage cervical cancer. Several studies have shown that both treatments are equally effective for patients with early-stage cervical cancer [36]. However, a systematic review by Yan et al. [37] suggested that patients at stage 1B1, 1B2, and IIA1 who underwent RH had a better survival rate than those who underwent CRT. The decision on which treatment to use is mainly determined by the patient’s health condition, age, and stage of the disease. Surgery is the best option in young people as it preserves hormonal and sexual functions. In elderly patients, who often have comorbidities, radiotherapy with or without chemotherapy is recommended [38]. Patients treated concurrently with RH plus adjuvant therapy have a higher risk of complications compared to CRT alone. For the past several decades, minimally invasive surgery (laparoscopic or robotic) has been the primary approach to the surgical treatment of early cervical cancer. The Laparoscopic Approach to Cervical Cancer Trial (LACC) prospectively compared laparotomic RH with minimally invasive RH (laparoscopic or robotic) and has significantly impacted the surgical treatment of early cervical cancer [39]. Disease-free survival (DFS) and overall survival (OS) were compared between the two groups. The 4.5-year disease-free survival rate was 86% for women who underwent minimally invasive surgery and 96.5% for those who had open surgery. Minimally invasive surgery was also linked to shorter overall survival. A retrospective study using data from the National Cancer Institute (NCI) Surveillance, Epidemiology, and End Results (SEER) project in the United States confirmed the findings of the LACC study, showing that minimally invasive surgery was associated with a higher mortality rate in cervical cancer patients compared to the laparotomic approach [40].

The ESGO (European Society of Gynecological Oncology) and NCCN (National Comprehensive Cancer Network) guidelines were updated to recommend laparotomic surgery as the gold standard method to perform a radical hysterectomy.

The risk definition given in the recently updated ESGO/ESTRO/ESP 2023 guidelines [16] is based on the following three prognostic risk factors: tumor size, maximal stromal invasion, and LVSI. These features divide patients with early cervical cancer into three risk categories. Low-risk patients are characterized by tumor size < 2 cm, no LVSI, invasion depth < 10 mm, and no lymph node involvement. Intermediate risk patients are identified by the “Sedlis criteria”, namely deep stromal invasion (above 1/3), lymphovascular space involvement, or tumor size > 4 cm. When two or more of these features are present, cervical cancer is classified as intermediate risk [41]. High risk patients are identified by the Peters criteria: positive LNs, positive margins, and/or microscopic parametrial involvement [42]. If positive pelvic lymph nodes, positive surgical margins, and/or positive parametrial involvement are identified, postoperative pelvic external beam radiotherapy (EBRT) with concurrent platinum-based chemotherapy is recommended (Table 2).

As per the latest revision of the European Guidelines for Cervical Cancer Treatment, published in 2023, simple hysterectomy (SH) represents the treatment of choice in patients with a TIA1 tumor regardless of lymphovascular spaces (LVSs) and in TIA2 LVS-negative tumors with negative margins. In this group of patients, no additional treatment is recommended other than oncological follow-up [16].

Conservative fertility-preserving therapies may be considered only after appropriate counseling for young patients with cervical cancer of <2 cm confined to the uterus (squamous cell carcinoma and HPV-related adenocarcinoma) who wish to have children. Procedures, such as conization or trachelectomy, may be performed based on the stage of the disease and related risk factors. Again, lymph node status assessment is a sine qua non for the fertility-sparing option.

Surgical lymph node assessment may be considered in TIA1 tumors with sentinel lymph node involvement and should always be performed in TIA2 LVSI-positive cases; the sentinel lymph node technique is considered an adequate treatment and can also be performed with minimally invasive techniques.

**Figure 2:**
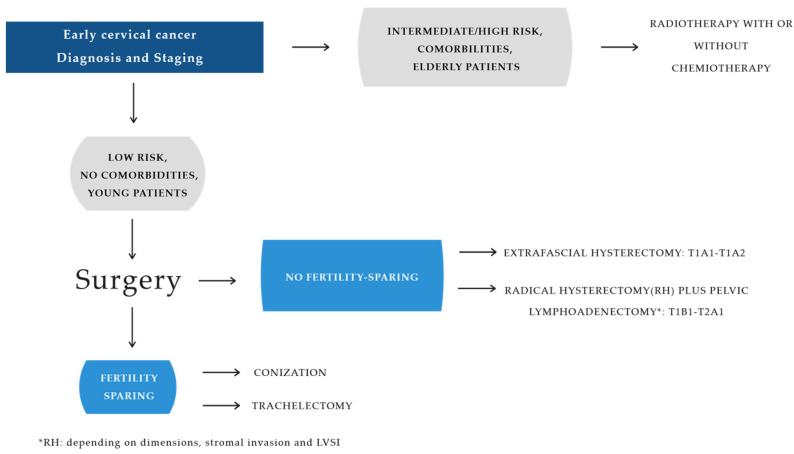
Early cervical cancer: diagnosis and staging.

Once lymph node positivity has been excluded intraoperatively by extemporaneous examination, radical hysterectomy (RH) with pelvic lymphadenectomy is the primary surgical approach for patients with stage TIB1, TIB2, and TIIA1 cervical cancer. The specific type of RH is determined by preoperative prognostic risk factors, including tumor size, stromal invasion, and lymphovascular space involvement (LVSI) (Figure 2).

With the aim of modulating radicality and preserving the main nerve pathways, in 2008, Querleu and Morrow introduced a new classification of hysterectomy, which was later updated in 2017 and that significantly revised the previous system established by Piver and Rutledge in 1974 [43,44]. The Querleu–Morrow classification, based on the extent of lateral resection, categorizes radical hysterectomy into the following four distinct types: RH A/B1 is reserved for low-risk patients, type B2/C1 is reserved for intermediate-risk patients, and type C1/C2 for is for high-risk patients. Additionally, type D (ultraradical) is dedicated to cases resistant to radiochemotherapy or recurrences of the lateral pelvic wall (Table 3).

The rationale for opting for a more radical parametriectomy stems from the need to remove any occult parametrial disease. While more extensive surgery improves survival rates and decreases the reliance on adjuvant radiotherapy, it comes with a higher risk of intraoperative and postoperative morbidity. Bladder dysfunction (ranging from 4% to 80%) and lymphocyst formation are among the most frequent complications following this type of surgery. Bladder dysfunction results from the sectioning of the ureter at the bladder base and the sectioning of the uterosacral ligaments, which disrupts the bladder nerves anatomically. Lymphocyst formation after radical hysterectomy with lymphadenectomy occurs due to disruption of the efferent pelvic lymphatics and can manifest as lymphedema, pelvic pain, and infections. The variation in the incidence of lymphocyst formation depends on the extent of the lymphadenectomy and whether or not a retroperitoneal drain is placed. The more radical the procedure, the more severe the neurological damage. Damage to the pelvic nerve fibers may also compromise intestinal function (about 40% of cases) and in over 20% of patients may also affect the sexual sphere with important repercussions related to the perception of one’s femininity. Deep vein thrombosis and pulmonary embolism may occur, but in recent years their incidence has decreased thanks to the use of antithrombotic prophylaxis with low-molecular-weight heparin administered from a few hours before surgery until complete resumption of ambulation and the use of antithrombotic stockings in the postoperative period. Recent evidence shows that magnetic resonance imaging (MRI) provides accurate preoperative information, thanks to its ability to define tumor diameter and stromal infiltration but, above all, its ability to safely assess parametric infiltration, fulfilling a key role in identifying the population for whom a less radical procedure is both safe and beneficial.

## 5. Discussion

Radical hysterectomy continues to be the standard of care for the treatment of early cervical cancer, but several research investigations have highlighted the need to consider the low-risk patient in a significantly different way than the intermediate- or high-risk patient with early cervical cancer [14,15,45,46,47,48].

Overall, the most pressing divergences to address in the patient with low-risk early cervical cancer are modulation of the radicality of the procedure, preservation of fertility, avoidance of parametriectomy with tumors less than 2 cm in diameter, the possibility of replacing bilateral lymphadenectomy with sentinel lymph node dissection, and persistence of MIC in place of laparotomy. All these findings are the result of the progress that has occurred in recent decades in the early diagnosis and staging of cervical cancer, made possible not only by the experience of surgical oncologists but, above all, by increasingly sophisticated imaging techniques that support the work of the clinician [49].

Accurate staging is, therefore, an essential component in the management of cervical cancer and preoperative imaging (T2W, DWI, ADC maps, and PET) is indispensable to ensure appropriate stage assignment and to identify the surgical patient.

### 5.1. Role of MRI in Assessing Prognosis

Accurately diagnosing low-risk disease prior to surgery is challenging. Only tumor size, which is related to prognosis, can be accurately assessed preoperatively by clinical assessment and MRI, while LVSI and depth of stromal invasion can only be determined after surgery on the anatomical specimen.

MRI is currently the best technique to measure tumor size, which is considered the most important prognostic factor, as it correlates with parametrial and lymph node involvement [25].

Two recent studies have shown that a tumor size greater than 20.5 mm is associated with deep stromal invasion, while a tumor size greater than 30 mm correlates with parametrial invasion. As a result, using cut-off values for primary tumor size, pretreatment measurements can be integrated into risk stratification models that may help guide therapy. Tumors smaller than 2 cm in diameter tend to have a better prognosis and are typically managed with surgery. These include stages TIA1, TIA2, and T1B1. TIA-stage tumors, depth of invasion < 5 mm, are not visible radiologically. The new classification focuses on the depth of invasion rather than the horizontal dimension, because it is more closely related to the risk of lymph node metastases [50]. The diagnosis is exclusively histological by conization, which is also based on the pathologist’s judgment on the lymphovascular space and positive margins. In patients with TIA stage 1 tumors, if fertility preservation is not desired, radical hysterectomy type A is the treatment of choice. This procedure involves an extra-fascial hysterectomy, where the paracervical tissue medial to the ureter is sectioned, and a small vaginal collar of less than 10 mm is removed. Surgical evaluation of lymph nodes may be considered in TIa1 tumors with LVS involvement and should always be performed in TIa2 LVSI-positive cases. The sentinel lymph node technique is considered an adequate treatment and can also be performed with minimally invasive techniques. Overall survival is between 70% and 90% [37].

For stage TIA2, radical hysterectomy type B is the treatment of choice. This procedure involves isolating the ureter and partially resecting the uterosacral and vesicouterine ligaments. Paracervical tissue is removed at the level of the ureter, with complete sparing of the visceral nerve fibers intended for innervation of the bladder. Colpectomy should be performed at least 10 mm from the tumor-affected tissue or cervix.

The earliest stage detectable by MRI is stage IB, where the tumor is confined to the cervix with deeper invasion greater than 5 mm.

In T2WI, a tumor > 5 mm appears hyperintense compared to the cervical stroma and the sequence allows a precise measurement of the nodule size, which must be provided in the following three planes: cranio–caudal (CC), antero–posterior (AP), and transverse (TS) [28]. The T2-weighted MRI predicts tumor size with a 93% accuracy versus 60% for clinical assessment [10,51]. An imaging marker known as the apparent diffusion coefficient (ADC) can be used as a quantitative parameter of DWI, offering valuable information about the density and aggressiveness of tumor cells [52]. In fact, carcinoma is hypercellular with a strongly limited tissue diffusion of water molecules so that carcinoma in DWI appears hyperintense compared to normal tissue with low values of the apparent diffusion coefficient (ADC). ADC measurement offers a quantitative assessment of the tumor; therefore, it is considered an imaging biomarker with several advantages, including non-invasiveness, no need for contrast agents, the absence of ionizing radiation, rapid acquisition, and easy integration into the clinical examination [31].

Once lymph node positivity has been excluded intraoperatively by extemporaneous examination, radical hysterectomy (RH) with pelvic lymphadenectomy represents the primary surgical treatment for patients with cervical cancer in stages TIB1, TIB2, and TIIA1.

The type of RH performed will depend on the preoperatively identified prognostic risk factors, namely tumor size, stromal invasion, and LVSI.

In patients with tumor volume > 2 cm, the tendency is to perform a radical hysterectomy type C1.

This type of surgery is characterized by the resection of the paracervical tissue starting from its lateral origin, represented by the emergence of the uterine artery from the obliterated umbilical artery, with sparing of the inferior hypogastric plexus and the visceral nerves responsible for the visceral innervation of the rectum and bladder.

Parametric infiltration must be excluded to confirm surgical treatment only. The presence of parametric infiltration defines stage IIB, and primary radio-chemotherapy is indicated [5].

The role of MRI is critical in the assessment of parametrial involvement. In these cases, sagittal T2WI shows an intermediate signal intensity lesion less than 20 mm in diameter within the cervix. The corresponding DWI and ADC map will exhibit associated restricted diffusion. Studies have reported that MRI, particularly with fused T2/DWI, demonstrates variable diagnostic accuracy in detecting parametrial involvement. The most effective parametric assessment is seen on axial oblique images, where the normal cervical stroma appears as a low T2 signal ring. If this ring is intact, it has a high negative predictive value (94–100%) for the absence of parametrial invasion [11]. Disruption of the stromal ring, along with visible nodular or irregular soft tissue extending into the parametrial area, suggests parametrial invasion. Post-biopsy cervical edema may mimic parametrial invasion. The use of DWI can help differentiate a post-biopsy edema from a tumor by identifying limited spread within the tumor [53].

A meta-analysis of parametrial invasion assessment revealed a specificity >90% for clinical examination (95% CI 83–89) and MRI (95% CI 90–95), but a sensitivity of only 40% (95% CI 25–58) with clinical examination compared with 84% (95% CI 76–90) with MRI [54]. Therefore, tumor size and parametrial invasion appear to be more precisely assessed with MRI.

### 5.2. Role of MRI in Reducing the Radicality of Surgery (Avoiding Parametriectomy)

The main reason for parametrial resection during primary cervical cancer surgery is to eliminate occult disease; in fact, approximately 30% of patients with early cervical cancer may have clinically undetected parametrial invasion [55]. The parametrium is adipose tissue containing blood vessels, autonomic nerve fibers, and lymphatics surrounding the cervix, so its resection can cause damage to the autonomic nerve fibers responsible for the innervation of the bladder, bowels, and sexual functions that run along the parametrium in 38% of cases, resulting in bladder and rectal dysfunction, sexual health problems (e.g., vaginal dryness), and fistula formation [56].

This underlines the importance of an accurate diagnosis of parametrial involvement and the need for partial resection of the parametrium while sparing the nerves that pass through it [12]. Several retrospective studies of patients with early low-risk cervical cancer who underwent radical hysterectomy have shown a very low rate (<1%) of parametrial involvement [15,45,48]; furthermore, randomized trials of nerve sparing (NS) RH versus RH have favored NSRH with regard to urinary function [57]. The poor parametrial involvement in low-risk patients and the morbidity that parametrial excision entails make the radical approach apparently unjustifiable [57]. The extent and utility of parametrial resection in radical hysterectomies in women with early cervical cancer have been widely debated in the literature over the past 15 years [46,47,48,49].

Numerous studies aim to investigate the possibility of reducing treatment-related morbidity without compromising oncological outcomes and question the need for parametrial resection in early cervical cancer [15,45,46].

Pluta et al. [58] conducted a study involving 55 patients who underwent complete pelvic lymphadenectomy and simple vaginal hysterectomy. With a median follow-up of 47 months, no recurrences were observed. The authors concluded that simple hysterectomy with pelvic lymph node dissection is both safe and feasible for selected women with early cervical cancer. Recently, the findings from two prospective studies, namely the Concerv study [59] and the LESSER study [60], have further reinforced the oncological safety of non-radical surgery in selected cases of early cervical cancer. In particular, in the prospective single-arm ConCERV study, patients with stage IA2/IB1 with low-risk features underwent simple hysterectomy and SLNB or conization with sentinel lymph node biopsy (SLNB) for fertility sparing.

The SHAPE study is the first large-scale, randomized, prospective trial to compare simple hysterectomy (SH) with radical hysterectomy (RH) in low-risk, early-stage cervical cancer patients [61]. The results indicate that, for low-risk, early-stage cases, simple hysterectomy does not affect oncological outcomes compared to radical hysterectomy, while resulting in fewer complications. However, further high-quality, prospective studies are necessary to confirm these findings. The SHAPE study provides long-awaited prospective evidence that simple hysterectomy is a safe alternative compared to radical hysterectomy for the treatment of low-risk, early-stage cervical cancer patients, with appropriate patient selection prior to less radical surgery being key. Conservative surgery, such as lymphadenectomy with simple hysterectomy or cervical conization, may, therefore, be an option for this group of patients [62,63,64,65,66] (Table 4).

### 5.3. RMI and the Preserving Fertility

Tumors smaller than 2 cm allow medical professionals to select cases suitable for fertility sparing (FS). MRI also plays a key role here. The joint ESGO-ESTRO-ESP guidelines [5] on cervical cancer recommend MRI as the preferred imaging modality to assess the suitability for FS in patients seeking to preserve fertility. Patients eligible for trachelectomy must be 40 years of age or younger, stage IA1 with LVSI, or stages IA2 and IB1, with MRI showing no parametrial invasion or metastasis to lymph nodes or other sites. Involvement of the internal os is a contraindication to trachelectomy [67]. Simple trachelectomy involves the extensive removal of the cervix, while radical trachelectomy includes the excision of the cervix, vaginal cuff, and parametrium, followed by an anastomosis between the isthmus and the vagina. Many experts suggest that preserving a significant portion of healthy stromal tissue during trachelectomy can help reduce the risk of complications, such as cervical incompetence, preterm labor, premature rupture of membranes, and other infectious conditions. Trachelectomy can be performed vaginally, abdominally via laparotomy, or laparoscopically. MRI is highly effective in evaluating internal os involvement, with a sensitivity of 91% and specificity of 97%. The distance between the cranial margin of the tumor and the internal os should be greater than 1 cm [68]. In sagittal T2WI, the internal os is identified as the narrowest point of the uterine body, where the low-signal cervical stroma transitions into the higher-signal uterine myometrium. The distance from the upper margin of the tumor to the internal os is measured in the sagittal plane. In terms of oncological safety, no significant difference in cure rates has been observed between radical trachelectomy and RH. An observational case–control study comparing vaginal or abdominal radical trachelectomy and RH for tumors up to 4 cm demonstrated similar recurrence-free survival and disease-specific survival rates, suggesting that oncological outcomes between the two procedures are comparable [69]. Fertility-sparing treatment is only viable for squamous cell carcinoma or HPV-associated adenocarcinoma when the tumor’s greatest diameter is 2 cm or smaller [16].

### 5.4. MRI: From Pelvic Lymphadenectomy to Sentinel Lymph Node Dissection

The presence of metastatic lymph nodes has a significant prognostic importance in early-stage carcinomas, with a survival rate of 90% in the case of negative lymph nodes and 65% in the presence of positive lymph nodes. Furthermore, preoperative detection of positive lymph nodes precludes surgery in patients with cervical cancer.

MRI has limited sensitivity for detecting lymph nodes (ranging from 17.2% to 59.1%), whereas PET is considered the most effective imaging technique for lymph node evaluation, offering higher sensitivity (73%) and specificity (98%) compared to MRI (56% sensitivity and 93% specificity) and CT (58% sensitivity and 92% specificity) [27]. Pelvic lymphadenectomy (PLND) is recommended for all patients with cervical cancer at stage IA1 with lymphovascular space invasion and for those at stage IA2 and beyond. The lymphadenectomy technique is related to the onset of intraoperative complications, such as hemorrhage, ureteral injury, nerve injury and postoperative complications, such as lymphocysts or lymph nodes based on the number of lymph nodes sampled during lymph node dissection. In 2015, to reduce the morbidity associated with pelvic lymphadenectomy, the National Comprehensive Cancer Network (NCCN) guidelines [70] stated that the sentinel lymph node (SLN) procedure should be considered as an alternative for PLND in cervical cancer. The sentinel lymph node technique is one of the most validated oncological techniques thanks to randomized, prospective, multicenter studies that have evaluated its diagnostic accuracy (Senticol 1, 2011 and Senticol 2, 2017), safety (SENTIX, 2020), survival, and QoL (SENTICOL 3, still ongoing) [71,72,73].

Sentinel lymph nodes are the first to receive lymph flow from the uterus and are detected with a sensitivity of 80–90% in the pelvic area, as demonstrated in many retrospective and prospective studies [71,74,75]. Sentinel lymph node negativity guarantees negativity of all regional lymph nodes with a negative predictive value of 100%, especially if bilateral [67]. In patients with negative sentinel lymph nodes, around 80–85% could potentially avoid complete pelvic lymphadenectomy and the associated morbidity. The technique can be performed laparoscopically, guaranteeing the patient minimal invasiveness and the pathologist the possibility of performing immunohistochemistry, arriving at a better characterization of the tumor but above all identifying micrometastases, which would otherwise go unnoticed and undetected with traditional histological analysis. The ability to identify micrometastases makes the sentinel lymph node the most sensitive technique for the diagnosis of nodal involvement.

Low-volume metastases (micrometastases and isolated tumor cells) are usually detected only by ultrastaging. After intraoperative processing, all SLNs are sent for ultrastaging. In the laboratory, they are stained with hematoxylin and eosin and undergo immunohistochemistry with anti-cytokeratin antibodies (AE1/AE3). Metastatic lesions measuring at least 2 mm in diameter are classified as macrometastases (MAC), while lesions ranging from 0.2 to 2 mm in diameter are considered micrometastases (MIC). Single cells or small clusters of cells up to 0.2 mm in diameter (less than 200 cells) are categorized as isolated tumor cells (ITCs). The 2018 FIGO classification [21] recognizes only macrometastases and micrometastases as significant lymph node metastases, while the presence of isolated tumor cells does not alter the staging. The SENTIX trial designed to evaluate oncological outcomes after SLN biopsy without pelvic lymphadenectomy has highlighted that sentinel lymph node mapping makes sense only if the procedure for ultrastaging is available, since sentinel lymph node biopsy alone reveals only about half of the cases of positive lymph nodes and does not reveal micrometastases; therefore, it is unreliable for intraoperative triage [74]. SLN ultrastaging instead has a potential decisional role in the indication of adjuvant therapies in the presence of micrometastases [73] (Table 5).

Numerous prospective multicenter clinical studies have been conducted that tend to demonstrate the non-inferiority and oncological safety of the sentinel lymph node technique compared to lymphadenectomy in patients with early carcinoma. The SENTICOL III study, which is still ongoing and whose results will be available in 2027, is studying the impact of sentinel lymph node biopsy alone on oncological outcomes in terms of disease-free survival and quality of life [76].

### 5.5. Persistence of MIS Instead of Laparotomy

Thanks to the Laparoscopic Approach to Carcinoma of the Cervix (LACC) study and other recent studies, the ESGO (European Society of Gynecological Oncology) and NCCN (National Comprehensive Cancer Network) guidelines have been revised to formally recommend open surgery as the gold standard method for performing radical hysterectomy. The demonstration that minimally invasive surgery (MIS) has a negative impact on the survival of patients with early-stage cervical cancer has erased the benefits claimed by MIS, such as improved quality of life and low rate of intra- and postoperative complications.

Studies by Kohler C suggested that the decrease in survival rates among cervical cancer patients treated with laparoscopy might be attributed to tumor manipulation and the potential spread of cancer during the procedure. In the case of smaller tumors, however, the uterus could be more easily handled, which may result in a lower likelihood of cancer spread [77].

Retrospective works by Margul DJ et al. and Melamed A et al. confirmed the results of the LACC study, but also reported that, in the subgroup of patients with tumors less than 2 cm in diameter, MIS did not result in a worse prognosis [40,78].

The study by Pedone Anchora et al. identified tumor size as the only pathological feature able to identify cases with different survival outcomes depending on the surgical approach [79]. The main determining factor in the choice of surgical approach is a tumor diameter of 20 mm. Women with tumors > 20 mm should undergo open surgery, while in case of tumors < 20 mm, both approaches appear safe. Therefore, laparoscopic surgery could still be considered an option in this subgroup of patients.

#### Advantages and Limitations

The primary strength of our review is the meticulous data collection method with well-specified article selection criteria. However, we acknowledge some limitations, including the retrospective design of the studies included in the review, with potential selection and publication bias.

## 6. Conclusions

MRI is advised as part of the initial diagnostic evaluation for the local staging of cervical cancer. Preoperative imaging is essential to ensure appropriate stage assignment and to develop more targeted treatment strategies in cervical cancer. Close collaboration between surgical oncologists and radiologists has led them to refine diagnostic capabilities over the years to the point of allowing appropriate patient selection and identification of the surgical patient without ignoring the importance of quality of life. The refinement of imaging diagnosis today allows researchers to explore surgical procedures that improve the quality of life without compromising oncological outcomes in low-risk patients by addressing various issues, such as reduced radicality, persistent MIS, and sentinel lymph node dissection, all measures that aim to increase survival, reduce morbidity, and, therefore, increase quality of life.

## Figures and Tables

**Table 1:** Comparison between categories, stages, and surgical–pathological findings with MRI findings and co-respective treatments [21].

Categories	Stages	Surgical–Pathological Findings	MRI Findings	Treatment
**T1**	**I**	Cervical carcinoma limited to the cervix (excluding extension to the corpus).	<5 mm, typically not visible on T2W images; dynamic imaging helps in detecting lesions up to 3–5 mm.	Surgery
**T1a**	**IA**	Invasive carcinoma identified only through microscopy, with maximum depth of invasion <5 mm.	Not visible on T2W images but can be detected on DCE.	
T1a1	IA1	Stromal invasion measured <3 mm in depth.		
T1a2	IA2	Stromal invasion measured ≥3 mm and <5 mm in depth.		
**T1b**	**IB**	Clinically visible lesion confined to the cervix or microscopic lesion larger than T1a.	Tumor seen on T2W and more clearly on DCE imaging, with an intact stromal ring. The tumor should be measured in terms of craniocaudal length.	Surgery
T1b1	IB1	Clinically visible lesion <2 cm in greatest dimension.		
T1b2	IB2	Clinically visible lesion ≥2 cm and <4 cm in greatest dimension.	Larger tumors may yield false-positive results.	Radiotherapy
T1b3	IB3	Clinically visible lesion ≥4 cm.		
**T2**	**II**	Tumor extends beyond the uterus but does not reach the pelvic wall or the lower one-third of the vagina.	Upper two-thirds of vagina.	
**T2a**	**IIA**	Tumor without parametrial invasion.	A positive “hypointense rim” sign on the axial T2W sequence indicates an intact stromal barrier	
T2a1	IIA1	Clinically visible lesion ≤4 cm in its greatest dimension.	False-negative results may occur in endophytic supravaginal tumors	Surgery
T2a2	IIA2	Clinically visible lesion >4 cm in its greatest dimension.	Larger tumors may result in false-positive findings.	Radiotherapy
**T2b**	**IIB**	Tumor with parametrial invasion.	Disruption of the stromal ring and parametrial invasion may be detected. False-positive results can arise from inflammatory lesions and pelvic congestion, which are considered a gray area.	
**T3**	**III**	Tumor extends to pelvic wall or involves lower one-third of vagina or causes hydronephrosis or nonfunctional kidney.	Thickening of the vaginal wall with altered signal intensity should be noted. Hidden areas, such as the fornices, must also be carefully examined.	Combination CT/RT
**T3a**	**IIIA**	Tumor involves lower one-third of vagina, with no extension to pelvic wall.	Lower one-third of the vagina.	
**T3b**	**IIIB**	Tumor extends to pelvic wall or causes hydronephrosis or nonfunctional kidney.	Tumor with 3 mm of pelvic sidewall/ureteric involvement.	
**T3c**	**IIIC**	Involvement of pelvic and/or para-aortic lymph nodes, regardless of tumor size and extent.		
T3c1	IIIC1	Pelvic lymph node metastasis only		
T3c2	IIIC2	Para-aortic lymph node metastasis		
**T4**	**IV**	Tumor invades mucosa of bladder or rectum or extends beyond true pelvis.	Bullous edema alone is not enough to classify a tumor as T4.	
**T4a**	**IVA**	Tumor invades mucosa of bladder or rectum.	Bladder/rectum involvement.	
**T4b**	**IVB**	Tumor extends beyond true pelvis	Distant metastases.	
**N1**		Regional nodal metastases, including the paracervical, parametrial, hypogastric, and iliac lymph nodes.	Node size > 1 cm or presence of necrosis.	
**M1**		Distant metastasis.	Distant organ metastases (such as to the lungs or liver) and lymph node metastases to the para-aortic region and above.	Palliative therapy

Table reproduced and updated according to the latest 2018 FIGO classification [21].

**Table 2:** Early cervical cancer risks categories.

Low Risk	Intermediate Risk	High Risk
Tumor size < 2 cm	Tumor size > 2 cm	Positive LNs
Invasion depth < 10 mm	Deep stromal invasion (>1/3)	Positive margins
No LVSI	LVSI involvement	Parametrial involvement
RH: A/B1	RH: B2/C1	RH: C1/C2

RH: Radical hysterectomy, LNs: lymph nodes, LVSI: lymphovascular space invasion.

**Table 3:** Querleu–Morrow classification of radical hysterectomy [43].

Type	Lateral Parametrium	Ventral Parametrium	Dorsal Parametrium
**A**	Located midway between the cervix and the ureter	Minimal excision	Minimal excision
**B1**	Within the ureteral bed region	Partial excision of the vesicouterine ligament	Partial resection
**B2**	Within the ureteral bed region plus paracervical lymphadenectomy	Partial excision of the vesicouterine ligament	Partial resection
**C1**	Transversely at the iliac vessels, with preservation of the caudal portion	Excision of the vesicouterine ligament at the bladder, including the proximal portion of the vesicovaginal ligament	At the rectum
**C2**	At the level of the medial aspect of iliac vessels, including the caudal part	At the bladder, sacrificing bladder nerves	At the sacrum, sacrificing the hypogastric nerve
**D**	At the pelvic wall, accompanied by resection of pelvic sidewall components and/or internal iliac vessels	At the bladder	At the sacrum

**Table 4:** More recent studies comparing radical hysterectomy (RH) to simple hysterectomy (SH).

Authors	Study	N° Patients	Stage	Type of Surgery	LFN	Follow-Up (Month)	N° Recurrences	Recurrences	
								RH	SH
Pluta, M. et al., 2009 [58]	Prospective study	55	IA2, iB1	SH	55	47	0	0	0
Wang 2017 [62]	Retrospective study	140	IB1	SH RH	140	75	3	2	1
Schmeler, K.M, et al., 2021 [59]	ConCerv study, prospective	100	IA2, IB1	SH conization	100	36	3	1	2
Liu Q. et al., 2021 [66]	Retrospective study,	440	IA2	SH RH	370	45	11	NR	NR
Carneiro, V.C.G, et al., 2023 [60]	Lessner study, prospective	40	IA2,IB1	SH RH modified	40	52	1	1	0
Plante M. et al. (2024) [61]	Shape study, prospective	700	IA2, IB1	SH RH	700	54	22	10	12

**Table 5:** The main studies on the oncological safety and quality of life of sentinel lymph node biopsy with or without pelvic lymphadenectomy.

Authors	Study	N° Patients	Stage	Objective	Results
Lecuru F, et al., 2011 [71]	Prospective **SENTICOL 1**	139	IA1,IB1,	Evaluated the diagnostic value of the SLN biopsy in patients with early-stage cervical cancer.	Bilateral negative sentinel lymph nodes accurately predict the absence of lymph node metastasis in early cervical cancer.
Mathevet P. et al., 2017 [72]	Prospective **SENTICOL 2**	206	IA1,IB1, IIA1	Compared the morbidity and quality of life after SLN biopsy alone and after SLN biopsy with PLN.	This study confirms that SLN alone is associated with reduced early morbidity and improved quality of life.
Cibula D, et al., 2020 [73]	Prospective **SENTIX**	647	IA1, IB2	Evaluated the oncological safety of SLN biopsy without additional pelvic lymphadenectomy in patients with early cervical cancer. Pathologic ultrastaging is a critical component of SLN biopsy in cervical cancer.	SLN ultrastaging detects an additional 43% of lymph node micrometastases in patients with negative LNs by imaging and intraoperative pathologic evaluation. As an international standard, ultrastaging should include examination of four levels of paraffin blocks, which detects >90% of patients with N1.
Lecuru FR. et al., 2019 [76]	Prospective **SENTICOL 3**	900	IA1,IB1	Aims to demonstrate the non-inferiority of SLN biopsy vs. SLN biopsy + PLN.	Still in progress.

## Data Availability

No new data were created or analyzed in this study. Data sharing is not applicable to this article.
